# Hazards of the job: absence of psychosocial risk in Occupational Health Medical Control Programs of slaughterhouses

**DOI:** 10.47626/1679-4435-2023-1063

**Published:** 2024-02-16

**Authors:** Bruna Carolina de-Quadros, Paulo Antonio Barros Oliveira

**Affiliations:** 1 Núcleo de Ergonomia e Capacitação em Segurança e Saúde Ocupacional da Engenharia de Produção, Universidade Federal do Rio Grande do Sul (UFRGS), Porto Alegre, RS, Brazil; 2 Graduate Program in Collective Health, UFRGS, Porto Alegre, RS, Brazil

**Keywords:** mental health, disease prevention, health strategies, worker health surveillance, saúde mental, prevenção de doenças, estratégias de saúde, vigilância em saúde

## Abstract

**Introduction:**

Workers from poultry and pork slaughterhouses have a higher frequency of sick leaves due to mental and behavioral disorders than the general working population.

**Objectives:**

This study aims to investigate how the Occupational Health Medical Control Programs of poultry and pork slaughterhouses deal with the psychosocial risk arising from working conditions.

**Methods:**

This observational-descriptive study of multiple cases is based on documentary research procedures and content analysis of 26 base documents of the Occupational Health Medical Control Program of slaughterhouses located in the state of Rio Grande do Sul, in October of 2017, with a quantitative-qualitative approach.

**Results:**

Only two slaughterhouses acknowledged the existence of psychosocial risks in their Occupational Health Medical Control Program. The study identified that only five companies developed some type of mental health strategy and those initiatives of mental health promotion and prevention of mental and behavioral disorders were classified as having low effectiveness. In their written programs, none of the 26 companies acknowledged that work can be a cause or a concause of mental and behavioral disorders.

**Conclusions:**

The non-recognition of psychosocial risk and the possibility of developing mental and behavioral disorders hinders the creation of adequate prevention and promotion actions, thus affecting the effectiveness of the Occupational Health Medical Control Program in terms of mental health preservation and burdening the Social Security system, due to sick leaves.

## Abstract (pt)

**Introdução:**

Trabalhadores de empresas de abate e processamento de carnes de aves e suínos apresentam frequência de afastamentos por transtornos mentais e do comportamento superior à da população trabalhadora em geral.

**Objetivos:**

Investigar como os Programas de Controle Médico de Saúde Ocupacional de frigoríficos de aves e suínos lidam com o risco psicossocial decorrente das condições e da organização do trabalho.

**Métodos:**

Trata-se de pesquisa observacional-descritiva de múltiplos casos, com o emprego de procedimentos de pesquisa documental e análise de conteúdo de 26 documentos-base do Programa de Controle Médico de Saúde Ocupacional de frigoríficos gaúchos, vigentes em outubro de 2017, com uma abordagem quanti-qualitativa.

**Resultados:**

Apenas dois frigoríficos reconheceram a existência de algum risco psicossocial em seus Programas de Controle Médico de Saúde Ocupacional. A pesquisa identificou que apenas cinco empresas desenvolveram alguma estratégia em saúde mental, sendo que essas medidas de promoção da saúde mental e de prevenção de transtornos mentais e do comportamento foram classificadas como de baixa efetividade. Em seus programas escritos, nenhuma das 26 empresas reconheceu que o trabalho pode ser causa ou concausa do desenvolvimento de transtornos mentais e do comportamento.

**Conclusões:**

O não reconhecimento do risco psicossocial e da possibilidade de desenvolvimento de transtornos mentais e do comportamento impede a elaboração de ações de prevenção e de promoção adequadas, afetando a efetividade do Programa de Controle Médico de Saúde Ocupacional quanto à preservação da saúde mental e onerando a Previdência Social, em razão dos afastamentos.

## INTRODUCTION

According to Regulatory Standard 17, adaptation of working conditions to workers' psychophysiological characteristics should meet requirements for comfort, safety and performance.^1^ Brazilian g working conditions include "aspects related [...] to workplace environmental conditions and work organization itself." However, from the perspective of ergonomic studies, it is known that work in slaughterhouses subjects employees to a range of recognized psychosocial risks, such as fragmented, monotonous, and repetitive work, imposed rhythm, low level of control, demand for high productivity, conflicting demands, and shift and night work.^2,3,4,5,6,7,8,9^

Despite suggestive findings in the specialized literature^3,7,8,10,11,12,13^ and the established epidemiological technical nexus, occupational physicians in the slaughtering industry and social security experts have not identified a relationship between mental and behavioral disorders (MBDs) and work.^5,6,14,15,16,17^ Since most secured leaves of absence consisted of sick leaves rather than accident-related leaves, in which there is an acknowledged nexus, the burden of disease is transferred to individuals, society, families, social security, and the Brazilian Unified Health System *(Sistema Único de Saúde,* SUS).

The aim of this study is to identity how mental health (MH) was approached in Occupational Health Medical Control Programs (Programas de Controle Médico da Saúde Ocupacional, PCMSOs) of the slaughtering industry, in view of the psychosocial risks that notably result from the working conditions in this industry. Based on this diagnosis, the present study proposes a reorientation in the planning of health promotion and primary prevention interventions.

## METHODS

This is an applied observational descriptive study of a case series based on the hypothesis suggesting inadequacy of policies for promoting health and for preventing distress and MBDs in slaughterhouse workers.

Herein, a mixed qualitative-quantitative approach was chosen to analyze data from the PCMSO of poultry and pork slaughterhouses located in the state of Rio Grande do Sul, Brazil, and controlled by the Federal Inspection Service *(Serviço de Inspeção Federal,* SIF) of the Ministry of Agriculture. Secondary data were supplied by the Division of Labor Inspection (Setor de Fiscalização do Trabalho, SEFISC) and by the Division of Occupational Safety and Health (Seção de Segurança e Saúde no Trabalho, SEGUR) of the Regional Superintendence of Work and Employment in Rio Grande do Sul (SRTE/RS) and covered documents produced by the companies from October 2016 to September 2017.

The variables of interest were retrieved from the PCMSOs through content analysis and organized in Microsoft Excel spreadsheets. By means of textual analysis of PCMSOs, we sought to identify the presence of absence of MH strategies and acknowledgment of psychosocial risks. The analysis of weaknesses and potentials of the promotion/prevention strategies identified implied exploratory research, with bibliographic research procedures and a qualitative approach of literature.

## RESULTS

Twenty-six documents were examined to find the terms related to the study, namely: psychy-, psych-, mental, distress, depress-, depr-, anxi-, and stress-. The words "distress," "depressed," and "anxious" did not occur in any of the documents examined. The radicals "psychi-" and "pshy" were often associated with the psychosocial assessment of workers involved in activities in confined spaces and high places, driving of automated vehicles, and firefighter crew members; moreover, specific psychiatric conditions, such as "anxiety," "depression," "bipolar disorders," and "schizophrenia", were only mentioned as factors that make workers unfit for these activities. There was no clear and direct mention that psychosocial risks arising from labor activity in slaughterhouses possibly cause anxiety, depression, or any other disorder or symptom.

Only two (7.7%) poultry slaughterhouses generically mentioned the existence of psychosocial risks in the activities developed. It is worth emphasizing that these two programs belong to companies linked to the same economic group, which controls another six units of analysis (base document of the PCMSO) that did not mention this type of risk.

None of the 26 programs assessed pointed MBDs as possible conditions associated with working conditions. Surprisingly given the low rate of acknowledged psychosocial risks and lack of indication of MH disorders related to work organization, MH promotion/prevention actions were planned or implemented in five companies, i.e., in 19.2% of the sample. Paradoxically none of the companies that acknowledged stress as a risk factor proposed a systematic MH promotion action.

## DISCUSSION

**Figure 1:**
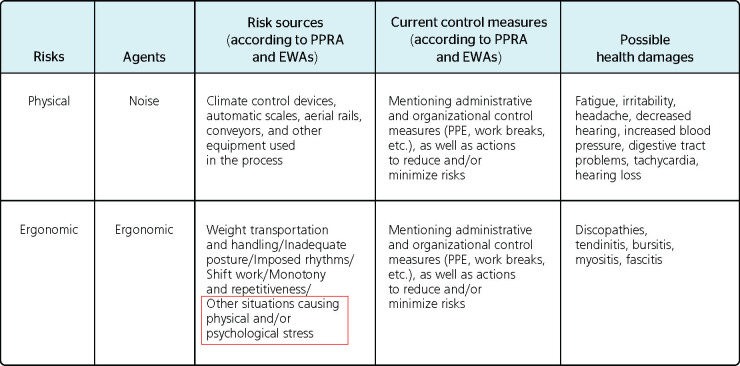
Generic mention of ergonomic risks arising from "other situations causing physical and/or psychic stress" without mentioning the corresponding possible health damage. EWA = ergonomic work analysis; PPE = personal protective equipment; PPRA = Environment Risk Prevention Program (Programa de Prevenção de Riscos Ambientais). Source: Occupational Health Medical Control Programs (Programas de Controle Médico da Saúde Ocupacional, PCMSO) of a poultry slaughtering plant in northwestern Rio Grande do Sul, Brazil.

**Figure 2:**
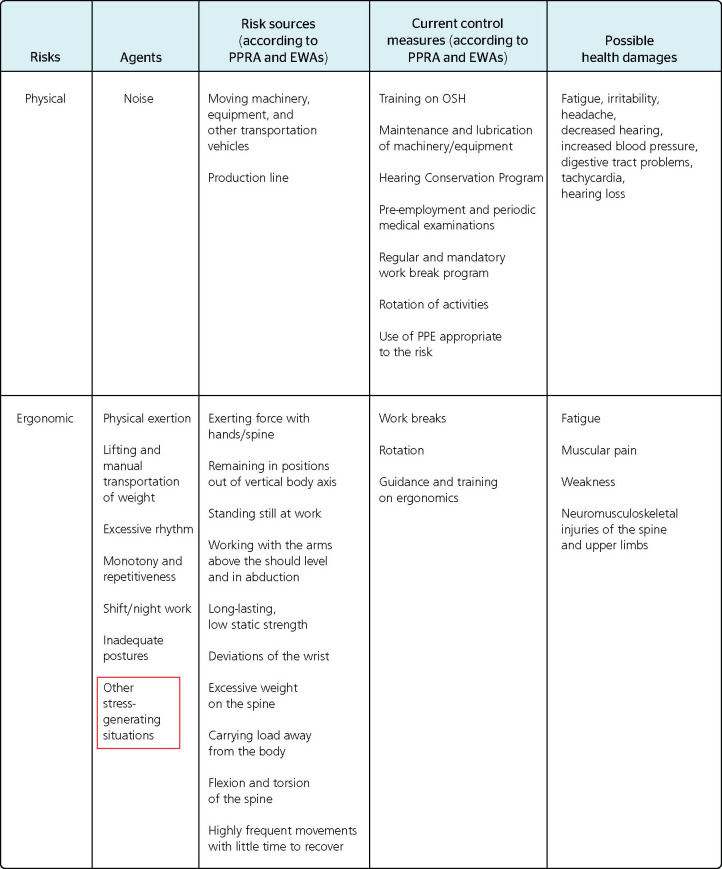
Chart mentioning "other stress-generating situations." EWA = ergonomic work analysis; OSH = occupational safety and health; PPE = personal protective equipment; PPRA = Environment Risk Prevention Program (Programa de Prevenção de Riscos Ambientais). Source: adapted from the Occupational Health Medical Control Programs (Programas de Controle Médico da Saúde Ocupacional, PCMSO) of a poultry slaughtering plant in the metropolitan mesoregion of Porto Alegre, Brazil.

It can be said that mention of psychosocial risk was superficial and generic, because, as shown in Figures 1 and 2, the documents did not present any indication of either the origin of situations generating risks for "psychic stress," current specific control measures, or MH disorders that may result from this risk.

In Figure 2, adapted from the PCMSO of another unit of the same economic group, there is a considerable improvement in the technical level of statements compared to those described in the previous figure. Despite mentioned as ergonomic risk agents, stress-generating situations, excessive rhythm, monotony, repetitiveness, and shift/night work have no parallel with the other items of the corresponding line.

Therefore, although the documents generically mentioned aspects that give cause to psychosocial risk, they did not identify the generating sources, the control measures adopted, or the health damages emerging from this risk. From the preventive point of view, the absence of these considerations makes it impossible to responsibly manage the risk arising from work organization. These inconsistencies were in line with the literature,^18,19^ especially with the analysis of the PCMSO of 30 companies from different economic sectors in the city of Salvador, Brazil, which evidenced the low technical quality of the programs.^20^

Employers shall inform employees about the risks and diseases to which they are exposed due to their labor activities (legal obligation provided under Art. 19, Paragraph 3º, of Law no. 8,213, of July 24, 1991, which sets forth that "the company shall provide detailed information on the risks of the procedures to conduct and of the products to handle"). Therefore, omitting possible health damages resulting from working conditions in the base document of PCMSO implies keeping workers unaware of the causal determinants and the relevant factors for their health-disease process. This situation leads to increased workers' vulnerability, since there is an inter-relation among "the quality of information people have, the ways how they retain this information, and the ability they have to incorporate it into their everyday practices."^21^ In this sense, it is very understandable that workers in the production line of slaughterhouses - such as those who working in the departments of slaughtering, cutting, and evisceration - have higher levels of depression, anxiety, mis adjustment, and vulnerability compared to administrative workers of the same slaughterhouses and to control groups.^8^

Conversely, from a health care planning point of view, the absence of any provision correlating MBDs to working conditions and organization hampers compliance with the normative objectives of the PCMSO, which aims to promote disease prevention and early screening.^20^ If there is no clarity on diseases, it is not possible to screen them.

Next, the weaknesses and potentials of the direct MH strategies identified in five slaughterhouses will be addressed. Among the actions implemented to promote MH and prevent distress, groups for prevention and control of psychiatric disorders were the most frequent ones.

### GROUP FOR PREVENTION AND CONTROL OF PSYCHIATRIC DISORDERS

Of the five companies that presented initiatives with possible direct effects on MH, only three organized a specific group to prevent and control psychiatric disorders. However, none of them admitted that working conditions could favor the development of MBDs.

The occupational health physician responsible for the PCMSO of two companies of the same geographic microregion in the state of Rio Grande do Sul, Brazil, provided for meetings with employees to offer educational, encouraging, and preventive guidance. He also considered the possibility of engaging psychologists and psychiatrists in health actions, in addition to allocating resources from the Mental Health Services (Centros de Atenção Psicossocial, CAPS) of employees' municipalities of origin. These actions would aim to identify, follow up, and advise employees under clinical treatment and/or those who had already presented a medical certificate but were not granted leaves of absence.

Similarly, the PCMSO of the third slaughterhouse provided for "monthly meetings held by a psychological support group, supported by the local government authority, which included workers with a medical certificate citing psychological problems." Although these meetings appear in the reports describing the health actions carried out in 2017, they are not mentioned in either the annual planning of PCMSO or in other passages of the base document of the program implemented by this poultry slaughterhouse, which employs 700 workers.

Therefore, we sought to identify whether the action strategies applied in the follow-up groups were clear and whether the tools and approaches adopted were well described. It was found that there was no rationale for the health action, since none of the three companies acknowledged psychosocial risks arising from working conditions and the psychopathological mechanisms acting in this professional context.

Consistent with the phenomenon of denial identified in the 26 PCMSOs evaluated, two action groups for preventing and controlling psychiatric disorders belong to a category that the PCMSO coordinator characterized as "non-occupational diseases with an epidemiological chronic-degenerative nature." Thus, the coordinator-physician excluded psychiatric disorders from list of occupational diseases, despite technical literature.

Restricting the participation in the groups to workers who were receiving or had already received clinical follow-up or who were granted medical certificates for health conditions arising from MBDs evidences the lack of a primary prevention strategy in the programs of the three companies. Prevention, screening, and early diagnosis, which should characterize the PCMSO, according to item 7.2.3 of NR 7,^22^ were not identified in the support groups of the three slaughterhouses described in this subsection.

Considering the duty of providing a balanced work environment, and due to the social role of property, it would be reasonable to assume that employers would apply their own resources to resolve the damage resulting from psychosocial risks. However, the study by Guilland & Moraes-Cruz^5^ reveals the impact of mental illness in slaughterhouse workers on social security finances, a fact that was also mentioned in the documentary entitled Carne, Osso.^14^ Hence, mental diseases of occupational etiology are little acknowledged in this type of industry; consequently, the social security financial burden is supported by society. In the three PCMSOs under discussion, it is possible to see a new attempt to socialize the losses generated by productive organization by means of transferring the responsibility and the costs of the treatment of workers with MBDs to SUS, through specialized care at CAPS or by other public healthcare professionals.

In view of the foregoing, the most evident weaknesses were: not including health actions in annual planning; restricting the target audience; lack of definition of clear strategies and intervention tools to be employed; low frequency of health intervention (once a month); lack of indicators or monitoring of the efficacy of health actions; dependency of public resources to intervene in a health-disease highly determined by occupation; and, finally not acknowledging that MBDs may result from labor activities.

One of the most important strengths of measures such as support groups is feeling that one belongs to a group, which may act as a protective factor for new crises, and sharing of positive coping strategies. To this end, it is necessary that these groups be facilitated and moderated so as to integrate the conflicting emotions arising from labor activity and from its impact on the other social roles played by workers in their families and in the community.

### INDIVIDUAL PSYCHOLOGICAL FOLLOW-UP

**Figure 3:**
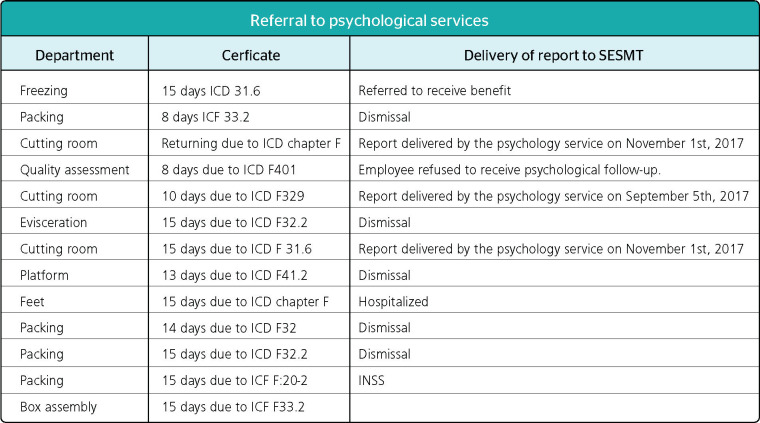
Psychological referrals in 2017. ICD = International Classification of Diseases; ICF = International Classification of Functioning, Disability and Health; INSS = Brazilian National Institute of Social Security (Instituto Nacional do Seguro Social); SESMT = Specialized Safety Engineering and Occupational Medicine Service (Serviço Especializado em Engenharia de Segurança e em Medicina do Trabalho). Fonte: health actions carried out in 2017 at a poultry slaughterhouse in the microregion of Caxias do Sul, northeastern Rio Grande do Sul.

In a poultry slaughtering house with more than 1,400 employees, 62 workers presented one or more medical certificates with International Classification of Diseases (ICD) chapter F in 2017. Of these workers, 21 were referred to psychological follow-up, which was not described or reported in the PCMSO. The company did not explain why only 21 (nearly one third) individuals were selected out of the 62 workers who presented medical certificates with ICD chapter F in the period. Moreover, employees suffering from diseases traditionally associated with slaughterhouse workers, such as recurrent depressive disorder, severe depressive episode, and anxiety-depression disorder, were dismissed, as shown in Figure 3.

Therefore, one of the perceived weaknesses is lack of directives to guide psychological referral/follow-up, since, based on the document analysis, there is a lack of clarity in the criteria adopted to select the workers to be referred for psychological care and in the employer s intention when adopting this measure. Additionally fear of being dismissed after psychological assessment may be an obstacle to the open talk that should guide a therapeutic relationship. Conversely it is important to ensure that mental health professionals acknowledge the ethical boundaries of their profession, especially the issue of psychologist-patient confidentiality and that they act according to this principle when communicating with the Specialized Safety Engineering and Occupational Medicine Service (Serviço Especializado em Engenharia de Segurança e em Medicina do Trabalho, SESMT) and with employers. Moreover, there were no indicators or monitoring of efficacy of this health action.

As for opportunities, it is possible to envisage the possibility of developing a well-structured program of psychological follow-up aiming to promote workers' quality of life and improve their mental health status, with great respect to their individuality and privacy. This program must be properly formalized, have clear referral standards that prioritize MH promotion and primary prevention without neglecting aspects related to rehabilitation, formulation of individual and collective coping strategies, and care through an individual therapeutic project. It is desirable that the program be presented to employees before their hiring and also in integration and refresher lectures, in order for all workers have enough information to protect their emotional integrity, considered here as an aspect of occupational health.

### ACTIVITIES PERFORMED BY PSYCHOLOGISTS LINKED TO SPECIALIZED SAFETY ENGINEERING AND OCCUPATIONAL MEDICINE SERVICE/HUMAN RESOURCES

**Figure 4:**
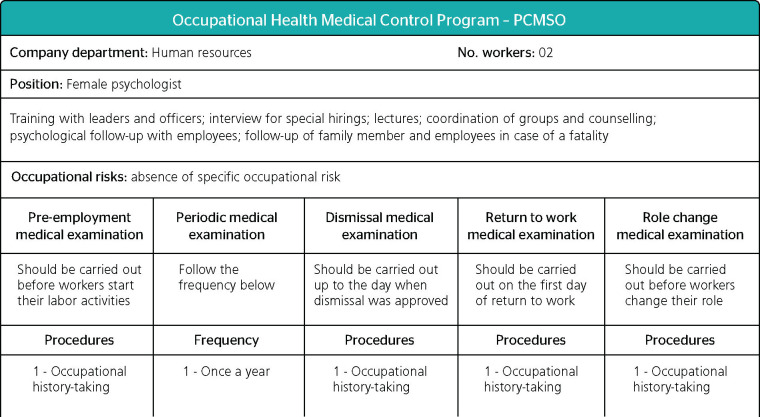
Chart describing risks and indicating psychologist's roles. Source: health actions conducted at a poultry slaughterhouse in the microregion of Guaporé, northeaster Rio Grande do Sul, Brazil, in 2017.

In the PCMSO of a poultry slaughterhouse with 1,200 direct employees and belonging to the same economic group of the slaughterhouse mentioned in the previous subsection, the chart of risks developed by the Homogenous Group of Exposure (HGE) lists the following psychologist's roles: a) training with leaders and officers; b) interview for special hirings; c) lectures; d) coordination of groups and counselling; e) psychologist follow-up with employees; and f) follow-up of family members and employees in case of a fatality (Figure 4). However, no chapter or item of the PCMSO addressed or regulated any of these activities.

There were no documents evidencing either the health actions implemented by the psychologist hired or the boundaries of the psychological follow-up supposedly provided. Similarly it was not possible to identify lectures palestras delivered or coordinated by psychologists among the health actions included in the Action Plan or evidence of these actions in other documents examined. If "fatality" is interpreted as a fatal accident, implying worker's death, occupational accident communications and accident reports with leaves of absence examined show no occurrence of this type of event, making it possible to infer the logical absence of psychological follow-up of family member and employees in the period analyzed.

From the mere description of the activities typical of the job position named "female psychologist" (noting that the gender cut is given by the PCMSO), the following weaknesses emerged: a) training with the psychologist was limited to leadership positions, not including the large population of workers in the production line; b) regular hirings (regular meaning ordinary common) are not subjected to psychological interview, limited to special hirings, who "special status" are not defined nor described in the PCMSO.

It is possible to envisage the opportunity of conducting interviews and psychological tests in order to delineate the desirable psychological profile and nontechnical skills that make workers less prone to distress and mental illness due to specific conditions and work organization. Although there are no studies on the topic, a possible self-selection bias may occur, predisposing workers with history or propensity to depression, anxiety, and other psychopathologies to the slaughtering job, since healthy workers would be more likely to obtain better positions in the labor market, in activities with more comfortable environmental conditions, without unwholesomeness, dirt, and daily and frequent contact with death.

Psychological assessment as a pre-employment requirement could prevent the hiring of workers who did not have personality attributes and interpersonal skills appropriate for the occupational risk identified in the slaughtering job. Although certain sanitation and process requirements are unavoidable, such as low and high temperatures and humidity, companies must control the risk according to the best technique, adopting appropriate collective, administrative, and individual measures and adapting working conditions to individuals, as dictated by ergonomics.

Still in relation to opportunities, expanding the training sessions provided by the psychologist to workers the production line, the so-called "factory floor" workers, would be an important action for the development and reinforcement of resilience and for the adoption of healthy (positive) coping strategies against stress and adversity. From the occupational health point of view, there is no sense in limiting training with this focus to those working in privileged hierarchical positions, since more ambitious and successful people perceive the challenges faced not as a threat but as a growth opportunity.

In view of the absence of justifications of the company to conduct trainings, it is possible that the training provided to officers and leaders focus on the development of interpersonal skills important to people management, such as good communication, assertiveness, leadership ability, and motivation. Undoubtedly, organizational climate would positively benefit from successful initiatives in this matter if there was coherence between official discourse (mission, vision, and values) and production management practice. Conversely, discourses inconsistent with practice have an opposite effect on workers' morale, give the schizophrenic nature of this type of paradoxical communication, characterized by double bind.^23^

### CAMPAIGN ON MENTAL HEALTH

In a poultry slaughterhouse with more than 700 employers which had a group to control psychiatric diseases, a campaign was identified in the same period with the theme "Well-being and psychological health," directed to all employees. According to the description included in the presentation sent to the Ministry of Labor, the campaign took place on only 2 days in August 2017.

The description of the event mentions leisure activities, plays, games, and stress day, with psychological guidance. On this occasion, workers were given a heart-shaped squeezing antistress ball.

Educational campaigns should be based on the most current knowledge on change management, organizational culture, and transformation paradigm. Despite being well-intentioned, campaigns that neglect important stages in the transformation process may be expensive and innocuous. As for the present case, the activity, which was not schedule in the annual planning of PCMSO, was carried out by the SESMT along with a consultancy company of the Brazilian S system. Although activities were directed to all employees, they took place only 2 days a year, which hinders the participation and engagement of the entire working staff, since they work in shifts. This campaign design does not allow for monitoring the efficacy of the measure.

It is also worth noting the inconsistency between the proposed activity and the assumed existing risks in the base document of PCMSO, since the company in question is not one of the two that had acknowledged stress as an ergonomic misadjustment. Therefore, a specific activity was proposed to combat stress, including the provision of a coping tool (little ball), in a company that does not acknowledge stress as a subproduct of its working conditions.

Educational campaigns are known to generate adherence if they are sufficiently prolonged in time and mobilize people towards organizational values and mission, aspects that are required to prevent damages to organizational climate and workers' health. The incompatibility of campaigns with the imperative structural conditions of the activity leads to resentment, sorrow, and other conflictive feelings,^23^ since production demands and safety are often contradicting.^24^

### ACTIONS ADDRESSING COPING STRATEGIES

This study also identified punctual, non-systematic actions of questionable effectiveness in terms of outcomes on workers' quality of life and well-being. Minor initiates such as those mentioned below may have positive results, but alone do not preserve workers' MH, which requires better structured, clear and conscious strategies.^25,26^

Actions that address or deal with coping strategies without naming them include: lectures about financial education, domestic, sexual and gender violence, child protection and family conflicts, risks of self-medication, and disease prevention; meditation workshops; groups dynamics promoting appreciation and self-esteem; implementation of motivational policies; and leadership trainings. None of the documents indicated that these strategies were applied with due transparency, i.e., previously clarifying that they are palliative promotion measures to deal with psychosocial risks not controlled otherwise.

Criminologists sustain that the harms caused to non-human animals are devastating not only to these animals, but also the human population, since slaughterhouse employment is significantly associated with increased total arrest rates, arrest for violent crimes, for rape, and for other sex offenses, after controlling for variables correlated with crime, such as proportion of young men, income levels, and immigration, among others.^27^

By reducing offender's compassion, violence against animals can increase tolerance or acceptance of violent attitudes and foster violence against humans,^28^ especially against the least powerful members of families and of society, such as women and children.^27^ Workers have difficulty in dealing with their emotions when kept in ignorance of the harmful effects of their professional activity, ignorance for which employers should not be excused. The social function of property imposes them to responsibly manage the risks arising from the way how they economically exploit the workforce for which they are responsible. Unfortunately, this study provides evidence in the opposite direction, pointing to neglect represented by lack of appropriate control and care measures.

One of the limitations of this study refers to the representativeness of the sample analyzed (26 poultry and pork slaughterhouses in Rio Grande do Sul, Brazil), which was not calculated with due rigor, thus hampering the extrapolation of findings. It is also worth noting the time elapsed from the development of the research (2018) and its publication as a complete article in 2022, 3 years after presentation in the 17^th^ Annual Congress of Brazilian National Association of Occupational Medicine and granting of Young Researcher award, in 2019.

## CONCLUSIONS

The lack of acknowledgment of psychosocial risk and of the possible development of MBDs logically hinders the development of appropriate prevention and promotion actions. This situation has an impact on the effectiveness of the PCMSO in terms of MH preservation and unduly burdens the Social Security System, due to sick leaves.

Initiative in MH identified in this work had unclear guidelines, with limited outreach and unmonitored efficacy among other listed weaknesses. The effectiveness of health promotion depends on the adoption of principles, such as a comprehensive concept of health, intersectionality empowering, social participation, equity, sustainability^29^ It is necessary to broaden social dialogue, which should start by acknowledging the risk emerging from working conditions in slaughtering plants.
